# Developing and psychometric testing of the evaluation tool for disaster exercises design stage: A mixed method study

**DOI:** 10.1371/journal.pone.0260581

**Published:** 2022-03-22

**Authors:** Hojjat Sheikhbardsiri, Mahmood Nekoei-Moghadam, Mohammad H. Yarmohammadian, Hamidreza Khankeh, Mohsen Aminizadeh, Abbas Ebadi

**Affiliations:** 1 Health in Disasters and Emergencies Research Center, Institute for Futures Studies in Health, Kerman University of Medical Sciences, Kerman, Iran; 2 Health Management and Economic Research Center, Isfahan University of Medical Sciences, Isfahan, Iran; 3 Department of Educational and Rehabilitation Psychology, Leipzig University, Leipzig, Germany; 4 Behavioral Sciences Research Center, life style institute, Baqiyatallah University of Medical Sciences, Tehran, Iran; Neijiang Normal University, CHINA

## Abstract

**Background:**

Exercise in different health sectors is an important step in preparing programs for disaster risk management. The present study aimed to develop and validate a tool for evaluating disaster exercises during the design stage in the health sector.

**Methods:**

This methodological study was conducted in two phases using an explanatory sequential mixed method approach. Semi-structured interviews with 25 disaster-related health professionals were conducted during the qualitative phase (item generation), and a systematic review was done to evaluate items pool of disaster exercises design stage tool. The quantitative phase (item reduction) assessed both face and content validity, as well as reliability using Cronbach’s alpha and intra-class correlation coefficient.

**Results:**

At the first stage four main categories and eleven subcategories were extracted from the data, the main categories including "coordination, command and guidance of exercise", "hardware and software requirements of exercise ", "organizational exercise resources", and "communication and exercise public information". The initial items pool included 164 items that were reduced to 50 after the assessment of validity (face and content). Cronbach’s alpha (0.89) and intra-class correlation coefficient (ICC = 0.72) tests indicated that the tool is also reliable.

**Conclusion:**

The research findings provide a new categorization perspective to understand the disaster exercises evaluation in the health sector. The existing 50-item tool can evaluate disaster exercises design stage in the health sector through collecting data with appropriate validity and reliability.

## Introduction

Disaster is a serious problem occurring over a short or long period of time that causes widespread human, material, economic or environmental loss which exceeds the ability of the affected community or society to cope using its own resources [1, 2].

Human societies have long been plagued by natural disasters, causing significant physical harm and death to a large number of people [3]. Today, disasters are on the rise around the world and have had a massive impact on human life and health, disturbing society’s capability to meet its fundamental needs and causing damage, inability and death of a large portion of the population [4].

Every year, an average of 354 natural hazards are recorded in the Emergency Events Database (EED), killing 68273 people and affecting 210 million people, with damages totaling more than $ 141 billion [2, 5]. Disasters cause small and large catastrophes that are sometimes difficult to recover from, depending on the type, scope, frequency, and density of the population exposed to the hazards [6]. Unpredictability is one of the most significant characteristics of natural hazards [7]. As a result, the only way to deal with this phenomenon is to be prepared, which can then prevent or reduce the amount of damage caused by it [7, 8]. The health systems, as one of the main pillars of the communities, play the most important role in disaster risk reduction [4, 9]. Disaster preparedness provides a platform for designing effective, realistic, and coordinated planning, reducing duplication of efforts and increasing its overall effectiveness and response efforts by National Societies, households, and community members [2, 10].

Krzysztof Goniewicz et al. concluded that disaster management qualifications should be connected to the hospital quality improvement process, with a focus on health worker in order to improve hospital preparedness [11]. Disaster exercises are the most important way to maintain and improve preparedness plans, and they can be used to test and validate policies, programs, and procedures, teach personnel, and improve inter-organizational communication and coordination [10]. Establishing and promoting specific functions codified in the response operational plan at different staff and operational levels of healthcare require the execution of operation-based and discussion-based exercises to identify and improve the strengths of the program and promote the relevant capabilities [12]. Exercise evaluation is an important step in designing and implementing exercise. It systematically investigates and indicates how well the exercise met its objectives. It also determines the strengths and weaknesses of the disaster exercise program [10, 13].

Jen Keynes et al. from Johns Hopkins University conducted a comprehensive study in 2009 that included a review of hospital preparedness in accordance with the National Incident Management System (NIMS). In this study, eight available instruments were used to assess the hospital’s preparedness: the Association for Professionals In Infection Control and Epidemiology (AIC), the Centers for Disease Control and Prevention (CDC), the Centers for Medicare and Medical Services (CMS), The Joint Commission (TJC), the Veterans Health Administration (VJHA), the South Carolina Department of Health (SCDH), the Texas Department of Health (TDH), and Agency for Healthcare Research and Quality(AHRQ). These instruments were examined with the principles of the NIMS and finally it was concluded that each of the existing instruments had defects and did not cover all the principles of NMIS [14]. Tang et al. conducted a study titled "developing an evaluation tool for hospital emergency preparedness in China." The researchers presented a methodology for evaluating hospital preparation drills in this study. The instrument is made up of 68 elements that assess structural and non-structural vulnerability, with 21 of them being structural. During a review of the tool, it was discovered that there was no item to evaluate the dimensions of hospital performance, and even the non-structural section only addresses a few dimensions, whereas modern preparedness assessment tools are expected to assess hospital capacity, service continuity, and the level of safety required to provide services by hospital staff. In addition, accurate information regarding the validation of the tool was not presented [15]. Mollie w et al. presented six evaluation modules to capture strengths and weaknesses of different aspects of hospital disaster response, including Predrill Module, Incident Command Center Module, Triage Zone Module, Treatment Zone Module, decontamination Zone Module and Group Debriefing Module. They also reported that these tools can evaluate specifics for general observation and documentation, victim tracking, biological incidents, and radiological incidents [16]. Nekoie-Moghadam et al. conducted a systematic review of the tools and checklists used to assess hospital disaster readiness and found that none of the reviewed checklists and tools addressed all of the elements required for a comprehensive hospital preparedness assessment [17].

One of the main obstacles to the accurate and scientific evaluation of exercises in the health sector is the lack of a standard and holistic tool for the evaluation of disaster exercises [18]. Many financial resources are spent in the health sector on disaster preparedness exercises. As there is no plan to control such exercises and they are not evaluated accurately to identify the strengths of organizations executing exercises, macro targeting and perspectives on health system preparedness in unexpected events have not been achieved, and they have not been measured scientifically [13, 19]. The above emphasizes the design and development of a comprehensive tool for evaluation of disaster exercises in the healthcare system in the exercise design phase to provide necessary protocols and instructions for evaluating disaster preparedness exercises. The present tool was developed to overcome the aforementioned obstacles and challenges, to evaluate accurately health exercises, and to increase the level of preparedness and the operational capacity of the health sector in disasters. The findings of this study also can be used as a guideline for developing standard preparedness exercises of the health system for emergencies and disasters.

## Methods

### Study design

This mixed-method study was carried out in two phases to design a comprehensive tool for the evaluation of disaster exercises design stage in the health sector. In the qualitative phase, interviews with Iranian disaster experts and systematic review were used for (Item Generation) and the quantitative phase was used for (Item Reduction) and validation of the measurement tool.

### Qualitative phase (Item generation)

To create the items, three major steps were taken: a) conducting interview with experts using directed content analysis to identify and develop the concept of disaster exercise evaluation, b) conducting systematic review and c) incorporating steps 1 and 2 for item pool.

#### Step 1: Qualitative study

This qualitative study was conducted in Iran, one of the most disaster-prone countries in the world. The study population included 25 experts who had practical experience or theoretical knowledge about "designing of preparedness exercises in disasters" and had participated in at least one operation-based or discussion-based exercise. A purposive sampling method with maximum diversity was used to select participants. Sampling was carried out until data saturation occurred, i.e. when the researcher concluded that additional interviews would not provide new information. Participants included seven pre-hospital managers, four hospital managers, two nursing experts in hospital emergencies committee, four experts in Emergency Operation Centers (EOC) in the Universities of Medical Sciences, six health experts in disaster risk reduction, and two vice chancellors of the logistic of the Universities y of Medical Sciences. The interviewees answered to a similar set of questions, which began with "have you ever experienced the disaster preparedness exercises of the health system?", "Describe the worst and best disaster exercises that you have experienced in the health system, "Based on your experience, what components and features should be considered in a standard discussion-based exercise? What components and features should be considered in a standard operation-based exercise? What requirements and functions should be considered when designing operation- and discussion-based exercises in disasters? Based on the above guide, additional questions were raised during the interview and when authors found new concepts. Moreover, for concept saturation, we use who, when, why and how as well as "Could you please give an example" or "Please explain more" for data and concept saturation. The interviews were recorded and lasted between 25 and74 minutes. The interviewer and interviewee agreed on the location and time of the interview; also, memos and notes were taken during the interviews to accurately describe and interpret the responses.

#### Step 2: A systematic review

The following step involved reviewing scientific studies and literature relevant to the research topic in order to add several items and complete the item pool. the papers searched using specific and relevant keywords in search engines and research databases, including ISI Web of science, PubMed, Scopus, Science Direct, Ovid, Pro-Quest, Wiley, Google Scholar and Persian database such as ISC, SID from January 1, 2000, to June 24, 2018. The following keywords and search strategies were used in conjunction with the Boolean operators OR and AND: (*simulation*, *practice*, *drill*, *exercise*, *design*, *instrument*, *tool*, *questionnaire*, *measurement*, *checklist*, *scale*, *test*, *inventory*, *battery*, *evaluation*, *assessment*, *appraisal*, *emergency*, *disaster*, *crisis*, *hazard*, *catastrophe*, *hospital*, *prehospital*, *health centers*, *treatment centers)*. This step was executed according to the PRISMA guideline [20].

*Inclusion criteria*. Persian and English articles evaluating disaster exercises in the health sector (both manmade and natural disasters), the articles addressing discussion- or operation-based exercises, articles describing the design of disaster exercises in the health system, and the articles addressing various techniques used to evaluate disaster exercises in the healthcare system.

*Exclusion criteria*. studies evaluating the performances of other organizations during disasters, including partner and support organizations such as firefighting, the Red Crescent, police and welfare studies evaluating the process of disaster risk management, including prevention and mitigation, response and recovery, studies presenting at conferences and proceedings, studies publishing in languages other than English and Persian before the year 2000.

#### Step 3: Incorporation of steps 1 and 2

The extracted items of disaster exercise were merged in the systematic and qualitative steps. The tables extracted from each study step were separated, and in this step, all components and characteristics get combined; redundant items get removed and similar ones get merged. Independent of the systematic review and qualitative study, new categories and subcategories get created. Since the new categories and subcategories served as the foundation for the pool of items, they get evaluated with greater sensitivity. The final table, containing the theme, category, subcategory, and codes expands the main exercise evaluation as converted into items. The research team examined questions and eliminated or modified several. Finally, an initial format for the disaster exercises design stage evaluation tool (DEDSET) prepared, consisting of 163 questions. Subsequently, the primary questionnaire’s validity determined. The psychometric properties of the DEDSET examined for face and content validity, as well as reliability.

### Quantitative phase (Item reduction)

#### Face validity

Ten experts experienced in disasters examined the tool’s dimensions and their relationships in order to determine the qualitative face validity of the items and analyzed the tool in terms of ease of completion, legibility, grammar, and the writing style of items in terms of ambiguity, level of difficulty and fitness. The research team re-read the phrases and incorporated experts’ suggestions. The item effect was used to quantitatively calculate the instrument’s face validity. For this purpose, participants assigned a value to each item using the five-point Likert scale ranging from five (quite important) to one (not important at all). Impact score = Frequency (%) ×Importance. The impact score was considered to be greater than 1.5 [21]. At this stage, no question was removed.

#### Content validity

For qualitative content validity to be ensured, ten health professionals experienced in disasters were asked to express their corrective views in terms of grammar, the use of appropriate words, proper placement of items, proper scoring and appropriateness of the selected dimensions. The questionnaire items revised in response to experts’ suggestions. Thus, most of the items transcribed by adding, substituting more common and understandable words, which led to the clarification of the vague items.

Content validity ratio (CVR) and content validity index (CVI) were used to evaluate the quantitative content validity. To begin, the CVR was calculated and a panel of experts was asked to rate each item on a three-point scale: necessary, useful, and not necessary. In this phase, the content validity ratio was calculated using the Lawshe formula (1975), which is acceptable with a score of 0.64 or greater [22]. CVR will be calculated using the following formula:

$$CVR=\frac{n_{E}-\frac{N}{r}}{\frac{N}{r}}$$

The criterion of "relevance" was used for each item on the one-point Likert scale to determine the content validity index. For this purpose, 10 experts were asked to determine the correlation between the questionnaire items according to the subscales of the questionnaire on a Likert scale ranging from one (not relevant) to four (completely relevant). Finally, K* will be calculated as follows, using the agreement ratio for the relevance of each item (I-CVI) and the probability of the chance agreement. According to Polit, the minimum number of evaluators required to calculate kappa using this method is three; the number of evaluators will be 10 in the present study. Kappa values of 0.59–0.40, 0.74–0.60, and > 0.74 will be considered poor, good, and excellent, respectively. In this study, only items with kappa of at least 0.74 will be accepted [23].


$$CVI=\frac{number\;of\;raters\;giving\;a\;rater\;3\;or\;4}{total\;number\;of\;raters}\;p_{c}=[\frac{N!}{A!(N-A)!}]\cdot 5^{N}\;k^{*}=\frac{I‐\mathrm{CVI}-p_{c}}{1-p_{c}}$$

#### Construct validity

In general, tools are classified as reflective and formative. In reflective tools, the items that make up the dimensions of the tool are conceptually related and structural validity is required to examine the dimensions of the tool. In formative tools, the items that make up the dimensions of the tool are not conceptually related [24]. Modern instrumentation for formative checklists precludes exploratory and confirmatory factor analysis [24]. In this study, different areas of health were involved in evaluating health system preparation exercises, including health, treatment, support, food and drug, so due to the different nature and functional areas of these sections in exercise, there was a lack of conceptual and functional relationship between items of components and eventually this tool had a formative nature.

#### Tool Reliability

Six operational exercises at Iran University of Medical Sciences were chosen for this study to test instrument reliability. Two evaluators evaluated each exercise independently. After the evaluators completed their evaluations of all exercises, the collected data was entered into SPSS 22 and the reliability of the instrument was examined using internal consistency and Intraclass Correlation Coefficient (ICC).

#### Ethical considerations

This study was approved by the Ethics Committee of Kerman University of Medical Sciences (IR.KMU.REC.1397.351). All participants voluntarily participated in this study. With the permission of the participants, their voices were recorded during the qualitative phase of the study. Names of the participants were not mentioned in the study, and instead of their names, codes were used in the interview texts. Other ethical considerations include explaining the research purpose and assuring the confidentiality of information to research units, voluntary participation in research, presenting research results verbally and in writing to research units and requesting a written informed consent.

## Results

### Qualitative phase (Item generation)

#### Step 1: Qualitative study

Table 1 shows how an original theme of the exercise design, four main categories, and eleven subcategories were formed. In the qualitative study, 382 codes were extracted from the concept evaluation of disaster exercises in the health sector.

**Table 1:** Categories and subcategories extracted from qualitative data.

Main theme	Categories	Subcategories
**Exercise design**	Coordination, Command and Conduction of Exercise	• Intra organizational and inter-organizational coordination • Design of an incident management system
	Exercise Hardware and Software Requirements	• Risk assessment • Incident operational plan • Consideration of upstream documents And consideration of lessen learning of past emergencies
	Exercise Organizational Resources	• Selection of competent personnel • Training of exercise personnel • Providing exercise financial and logistic resources
	Communication and Exercise General Information	• Providing exercise information resources • Providing of communications infrastructure • Providing cultural and social priorities

#### Step 2: Systematic review

The initial search of the literature in electronic databases yielded 5578 documents. The number of documents decreased to 2789 articles after duplicates, books, dissertations, and presentations were filtered. First, the titles and abstracts were reviewed to find those related to the evaluation of health exercises for emergencies and disasters and 123 eligible articles were extracted based on systematic screening. Then, all 123 selected full‑text papers were reviewed, and finally, 10 papers reported evaluation of health preparedness exercises for emergencies and disasters. In a systematic review, 203 codes of the components of evaluation of health preparedness exercises were extracted from 10 related articles.

#### Step 3: Incorporation of steps 1 and 2

After two meetings with the research team and professionals, the third step consisted of removing repeated items, merging similar ones, and finally, reducing the number of items to 63 by selecting the best relevant items, followed by the psychometric process.

### Results of the second phase of the study

#### Face and content validities

To begin, four items were corrected for spelling and no items were removed during the qualitative face validity stage. Following the calculation of the impact score, 63 items with impact scores ≥ 1.5 were considered favorable and kept for further analysis in the quantitative face validity stage.

In order to assess the qualitative content validity, most of the items were transcribed by adding, reducing or replacing some words with more common and understandable words, which led to the clarification of ambiguous items. Items with the same implications were removed and some of them were merged because of overlapping. At these stages, 8 items were merged into other items, and 55 items were kept for further analysis. Based on the opinions of 10 experts, the CVR was considered significant during the quantitative content validity stage (> 0.59). Thus, three items were removed. At the end of this stage, the number of items in the questionnaire was reduced to 52 ones. According to Polit, two items with Kappa coefficients > 0.74 were omitted [23], bringing the total number of items to 50. Then, based on the mean CVI scores of all items, the mean CVI of the whole tool was calculated, with 0.9 being the acceptable standard [25]. Table 2 shows that 0.97 obtained in the present study is acceptable.

**Table 2:** Results of validity of measurements.

Item	Impact score	CVR	CVI	K
1- The exercise executors have obtained official permission to perform the exercise from the highest person in charge of the organization.	4.7	0.8	1	1
2- Before the exercise, the necessary consultations were held with intra- and extra-organizational units that had previously held the exercise.	4.5	0.8	1	1
3- To coordinate the level of exercise, meetings with other intra- and extra-organizational units were held during the design of the current exercise.	4.5	1	1	1
4- The response plans of other organizations participating in the exercise were taken into account when designing the exercise.	5	0.8	1	1
5- Depending on the level of exercise and documentation, an intra- and extra-organizational agreement with disaster/event management units is available.	5	0.8	0.8	0.78
6- The incident operation plan specifies the positions and roles of intra- and extra-organizational units participating in the exercise.	4.9	1	1	1
7- The chart of the incident command system has been designed in all units of the healthcare, including health, treatment, support, education, student-culture, and food and drug.	5	1	1	1
8- The person in charge and his successors have been specified for all positions in the incident command system.	**3.4**	**0.8**	**0.70**	**0.66**
9- The responsible individuals, their successors, job descriptions in different positions of the incident command system as well as the standard response schedules of 0–2, 2–12 and more than 12 specific hours have been defined.	4.1	0.8	1	1
10- The executive structure of the exercise has been designed according to the position of the organization at local and national levels, and the job description of the relevant committee has been specified.	4.3	0.8	0.9	0.98
11- Risk assessment and hazard prioritization have been performed using the national hazard assessment tool and the present exercise has been designed accordingly.	3.4	0.8	0.9	0.98
12- Before the exercise, the risk map for all preferable hazards was created and drawn in the EOC of the executing organization.	4	0.8	0.9	0.98
13- An early warning program for preferable hazards has been designed in the EOC based on the national leveling program.	4.3	1	1	1
14- The exercise scenario has been precisely designed and provided to intra- and extra-organizational units based on the hazard prioritization of the organization.	3.6	1	1	1
15- The present exercise was planned based on needs assessment, a review of previous incidents in the area in question.	3.5	0.8	0.9	0.98
16- The incident operation plan was designed and developed based on the national model approved by the secretariat of the working group for the implementation of the exercise.	4.9	0.8	1	1
17- The present exercise has been designed by studying and considering the upstream documents, including the national exercise program in the Iranian health system.	5	1	1	1
18- The exercise’s objectives make it clear which aspects of organizational performance will be evaluated.	3.5	0.8	1	1
19- Planning for the preparation of the exercise schedule has been provided, from the pre-design phase to the stage of modifying and upgrading the response operation plan.	5	1	0.9	0.98
20- The exercise-planning group has prepared a list of the main functions and activities that are expected during the exercise.	5	1	1	1
21- The present exercise was designed by an expert group experienced in disasters or by consultation with the mentioned people.	5	0.8	1	1
22- Representatives from the local community, partner and support organizations attended in the detailed and general exercise design meetings of the organization.	4.4	1	1	1
23- Discussion-based exercise (workshop, seminar, game, table-top exercise) was considered and performed in accordance with the exercise objectives before the operational exercise.	4.6	0.8	1	1
24- The locations of the discussion- and operation-based exercises were selected according to the type and level of the exercises included in the scenario and based on scientific principles.	4.7	0.8	1	1
25- All participants have given their informed consent to participate in the present exercise and all necessary documentation is in place	4.6	0.8	0.9	0.98
26- The organization’s EOC has a public safety and security response plan in place, which includes initial warnings, timely behavior, and incident response.	5	0.8	1	1
27- There is a program on how to call the employees of the responsible organization and how to employ the voluntary staff, partner and support organizations to participate in the exercise.	3.4	0.8	1	1
28- The organization’s EOC has a program to assess the competence of the employees of the units participating in the exercise.	**4**	**0.4**	**-**	**-**
29- The job action sheet (JAS)has been prepared for the people participating in the exercise to familiarize them with their roles and responsibilities.	4.6	1	1	1
30- The budget and financial resources necessary for the different phases of the exercise have been predicted in the financial statement row of the organization executing the exercise.	5	0.8	0.9	0.98
31- Support services, including the provision of ID cards or etiquette, cooling-heating equipment, communication, nutrition, parking, simulated locations, transportation, sanitary services and other requirements have been prepared for all staff participating in the exercise.	3.4	0.8	1	1
32- Appropriate means of transportation have been provided to transport resources to the exercise location.	**4**	**0.4**	**-**	**-**
33- The EOC of the organization has been outfitted with communication equipment, physical space, work force and necessities in accordance with the health response program (developed by the working group).	4	0.8	0.9	0.98
34- The members of the rapid health assessment team are available in terms of the number of experts and their contact numbers based on the level of the incident determined in the exercise scenario.	4.7	0.8	0.9	0.98
35- The equipment and supplies required for the disaster medical response group are ready and available	**4**	**0.4**	**-**	**-**
36- The exercise evaluation group has been specified, and the team leader has provided the necessary training on how to evaluate the exercise.	5	0.8	0.9	0.98
37- Exercise evaluators have the necessary expertise and experience to evaluate the exercise in accordance with the type and objectives of the exercise.	5	1	1	1
38- In accordance with the evaluation and promotion of the national exercise program group and the functional dimensions, possibilities included in the scenario, an appropriate number of exercise evaluators have been considered.	5	1	1	1
39- Participants in the table-top exercise have the necessary expertise and experience in the field of disaster exercises.	3.8	1	1	1
40- Experts experienced in disasters were chosen as instructors and lecturers of discussion-based exercises, including workshops and seminars.	4.2	1	1	1
41- A specific person has been intended to document the whole process of exercise as well as the lessons learned.	5	1	1	1
42- Controllers, facilitators, actors, and evaluators have received specific training prior to the exercise.	4	0.8	1	1
43- In accordance with the objectives of the exercise, necessary written measures have been predicted to ensure the safety and security of the participants (prediction of the necessary interventions in case of a real incident during the exercise).	3.4	0.8	1	1
44- The members of the medical-relief response team and their job descriptions have been specified.	**4.1**	**0.8**	**0.6**	**0.49**
45- The exercise staff have the necessary physical and mental abilities (physical fitness), as well as motivation, seriousness, and desire to participate in the exercise.	2.34	0.8	0.9	0.98
46- There is a complete and up-to-date list of equipment and supplies needed to perform exercises at different levels in the organization.	4.3	0.8	1	1
47- The operations center has updated contact information for other centers and organizations related to disaster exercises	3.8	0.8	0.9	0.98
48- A comprehensive database of all members of the organization participating in the exercise has been prepared including telephone numbers, contact methods and addresses.	5	1	1	1
49- The EOC of the organization has a suitable communication program with different and multi-layer platforms ranging from command to operation levels.	4	1	0.9	0.98
50- The equipment required for communication, including telecommunication between intra- and extra-organizational units participating in the exercise has been provided.	3.8	1	1	1
51- Cultural factors (including the observance of Islamic manners, the language and culture of the community where the exercise is executed) have been considered in the process of exercise.	4.5	0.8	1	1
52- The coordination meeting, training program, and documentation for media participation in the exercise process are available.	3.2	0.8	1	1
53- The local media has provided the necessary public information about the goals, process and location of exercise to prevent intimidation, terror, rumors and disturbance in the public order of the society.	5	0.8	1	1
54- Necessary coordination has been done for the participation of the documentary group (film and photo) in different locations of exercise as one of the evaluation techniques.	2.59	0.8	0.9	0.98
55- Necessary measures have been taken to prevent very important person (VIP) from entering the exercise location and causing disruption during the exercise.	4.8	0.8	1	1
			S_CVI/Average = 0.97	

#### Tool reliability results

Internal consistency of the dimensions of the tool for evaluation of disaster exercise design was more than 0.7 and the internal consistency of the whole tool was 0.89, indicating high reliability. ICC was also used to calculate the stability of the tool, which was higher than 0.75 for all questions. ICC of the whole tool was 0.90, indicating that the DEDSET was reliable (Tables 3 and 4).

**Table 3:** Determining reliability with internal consistency (Cronbach’s alpha) in terms of dimensions of the evaluation tool of disaster exercise design in healthcare sector.

Exercise design			
The Tool Dimension	The number of items in Each Dimension	Cronbach’s alpha	Mean ± SD
Coordination, Command and Conduction of Exercise	8	0.719	16.00 ± 3.74
Exercise Hardware and Software Requirements	21	0.926	48.33 ± 10.44
Exercise Organizational Resources	11	0.928	26.00 ± 5.42
Communication and Exercise General Information	9	0.908	18.58 ± 4.39

**Table 4:** Determining the reliability with ICC in terms of dimensions of the evaluation tool of disaster exercise design in healthcare sector.

Exercise phase	Dimension	Item No.	ICC	Mean ± SD
Exercise design	Coordination, Command and Conduction of Exercise, Exercise Hardware and Software Requirements, Exercise Organizational Resources, Communication and Exercise General Information	50	0.72	117 ± 19.85

#### The final tool

The checklist for evaluation of exercise design in the healthcare system includes 50 items divided into four dimensions of "Coordination, Command and Conduction of Exercise", "Exercise Hardware and Software Requirements", Organizational Resources of Exercise "," Communication and General Informing of Exercise ". The checklist items were weighed and scored based on the opinions of the experts experienced in disaster response in Iran. The checklists are scored based on the functions that are expected to be performed in the stage of exercise design.

- Mark No. 2 if the expected function was performed correctly and on time.
- Mark No.1 if the expected function was performed, but its quality or timing was improper.
- Mark No.0 f the expected function was not performed.

#### The cutoff point of the tool

According to the total scores of 50 items on the three-point Likert scale, the maximum and minimum scores were 100 and 0, respectively. In this tool, scores 0–33.5 indicate poor preparedness, 33.6–66.5 indicate moderate preparedness, and 66.6–100 indicate good preparedness. In addition, the level of preparedness of each health sector is calculated and interpreted in terms of percentage by using the linear transformation formula, converting the tool’s score into a percentage and comparing it with the maximum and minimum scores of the tool. Poor preparedness is represented by 0–33.5 percent, medium preparedness is represented by 33.5–66.5 percent, and good preparedness is represented by 66.5–100 percent.

## Discussion

Coordination, command and conduction of exercise, exercise hardware and software requirements, organizational exercise resources, communication and general exercise information are the main components affecting the design of preparedness exercises in health system. The findings of this study indicated that coordination, command and conduction of exercise were some of the foundations for designing discussion-based and operation-based exercises of the health system in disasters. This finding is consistent with findings of the studies [26, 27]. Other studies have shown that in order for an organization to be fully prepared to respond effectively to an incident by conducting exercises, all constituent organizational units must synchronize and coordinate functions [28, 29].

According to the findings, the main foundation of the exercise program in the design phase is the provision of hardware and software requirements before executing the main exercise. Issues such as risk assessment and prioritization of hazards, provision of a risk map, operational incident plan, early warning program, exercise scenario and execution of a discussion-based exercise before an operation-based exercise should be considered during the design phase of the exercise. Various studies suggest that risk assessment determines the nature and extent of the risk and is based on the analysis of potential hazards as well as the vulnerability of the property, livelihoods and environment involved with risk or potential harm [30, 31].

One of the factors explaining the concept of the exercise design was the provision of organizational resources for exercise. This finding is consistent with findings of the studies [32, 33]. Regarding the preparedness of health organizations through design and implementation of exercise, enhancement of the organizational efficiency is dependent on increasing the human resource efficiency, which in turn is dependent on developing knowledge, skills and creating appropriate behaviors for successful performance in disasters.

Communications and public information on exercise was one of the main categories of interviewing with health experts. According to some research, one of the requirements of organizational preparedness in disasters is the ability to predict appropriate communication between organizations and important locations [34, 35].

There are various tools in the world for the evaluation of disaster exercises in hospitals as part of the healthcare sector. These evaluation tools have been presented as activity-based checklists, but there was no fundamental macro-level tool for all health departments, including treatment, health, support, food and drug to design different types of exercises. For example, Heidaranlu et al. [36] designed and validated a tool for evaluation of the functional preparedness of the hospital in natural hazards. When this tool was compared to the one used in our study, it was discovered that while they were similar in the validation process, they were different in construct validity. Our tool requires no exploratory factor analysis (EFA) or confirmatory factor analysis (CFA) because of the study’s formative nature. The tool mentioned above is used to evaluate the functional preparedness of hospitals, but our tool specifically evaluates the functional preparedness of the entire healthcare sector to respond to disasters through evaluation of the exercise design stage.

Cheung et al. [37] wrote a book called Hospital Preparedness Exercise: Atlas of Resources and Tools. The book covered topics such as exercise design, execution and evaluation, as well as a review of resources available for hospital functions. The book only provided sources for available evaluation tools of hospitals, with no information on the tools’ items, the validity and reliability.

Tang et al. conducted a systematic review of available preparedness tools in their study titled "Development of a tool for evaluation of hospital emergency preparedness in China". In this study, they presented a tool for the evaluation of hospital exercises. The tool did not include any items to examine the functional dimensions of the hospital, and it only dealt with a few dimensions. Preparedness evaluation tools are expected to assess the capacity of hospitals, services and the level of safety required for hospital staff. In addition, they did not provide in-depth information on the validity and reliability of the tool developed in their study. The main limitation of the studies selected in the final analysis for tool development, according to the researcher, was a lack of validity and reliability of the tools [15]. The current tool was developed in the Persian language and then translated into English. Thus, non-Persian speaking researchers validated the translation.

## Limitation

This study had several limitations. As our study, samples were disaster exercises in the healthcare system, coordination for the design and execution of disaster exercises required significant costs and the overcoming of the maze of administrative hierarchies in the research quantitative phase for performing tool reliability. Some operational exercises were done with the help of the research team and communication with the Emergency Operations Center (EOC) in Iran’s health ministry in order to solve this problem. The second limitation was the lack of factor analysis with varimax rotation of the items to improve definition of each measurement in the categories and subcategories. Therefore, further research and investigation are needed for advanced validation.

## Conclusion

The study’s tool was developed as a result of interviews with twenty-five disaster professionals in health field, as well as a literature review and validation procedure. Our study tool includes the most important indicators and dimensions required for disaster exercises design in the health sector, including exercise coordination, command and guidance, exercise hardware and software requirements, organizational resources of the exercise, and communications and general informing of the exercise. Therefore, the present tool can play an important role in increasing the level of preparedness and improving the operational capacity of health and treatment against disasters by evaluating accurately the disaster exercise design stage in the healthcare sector.

In this study, we only did the preliminary validation for the developed tool, it is recommended that a study on the implementation of construct validity should be conducted to complete the validation process.
